# Glycation of LDL: AGEs, impact on lipoprotein function, and involvement in atherosclerosis

**DOI:** 10.3389/fcvm.2023.1094188

**Published:** 2023-01-24

**Authors:** Anastasia V. Poznyak, Vasily N. Sukhorukov, Raisa Surkova, Nikolay A. Orekhov, Alexander N. Orekhov

**Affiliations:** ^1^Institute for Atherosclerosis Research, Moscow, Russia; ^2^Laboratory of Angiopathology, Institute of General Pathology and Pathophysiology, Moscow, Russia

**Keywords:** LDL, LDL modification, atherosclerosis, lipids, AGE, glycation

## Abstract

Atherosclerosis is a complex disease, and there are many factors that influence its development and the course of the disease. A deep understanding of the pathological mechanisms underlying atherogenesis is needed to develop optimal therapeutic strategies and treatments. In this review, we have focused on low density lipoproteins. According to multiple studies, their atherogenic properties are associated with multiple modifications of lipid particles. One of these modifications is Glycation. We considered aspects related to the formation of modified particles, as well as the influence of modification on their functioning. We paid special attention to atherogenicity and the role of glycated low-density lipoprotein (LDL) in atherosclerosis.

## 1. Formation of advanced glycation end products

AGEs represent a group of heterogenous molecules, the common features of which are covalent cross-link formation among proteins, the effect of transforming the color of some food products into yellow-brown colors (“browning” effect) and fluorescence formation (1). Regarding their characteristics, AGE can be divided into two groups: AGE with fluorescence and cross-links including pentosidine, crossline, 2-(2-furoyl)- 4(5)-(2-furanyl)-IH-imidazole, glyoxal–lysine dimer, and methyl- glyoxallysine dimer (MOLD); and AGE without fluorescence (non-fluorescence) and cross-links including N ^ε^-(carboxymethyl) lysine (CML), N ^ε^-(carboxyethyl)lysine (CEL), and pyrralline (2).

The reaction, which produce the advanced glycation endproducts (AGE) is known as the Maillard reaction. It is a typical non-enzymic browning reaction between the free carbonyl group of a reducing sugar and the amine group of a protein. It was first described in 1,912 and demonstrated the effects of AGE on food caused by storage and heat processing. The similar process within the human body was described only about 50 years ago (3). Maillard reaction occurs in three stages. The reaction initiates with the covalent bound of the reducing sugar to an amine group of protein. Arginine, sulfur, and lysine-containing amino acids are especially prone to glycoxidation while fructose, glucose, mannose, galactose, ribose, and reactive triose intermediates participate in the endogenous formation of AGE. The condensation of a carbonyl group of sugar with an amine group of protein forms an unsteady Schiff base adduct or glycosylamines in hours. After that, the Schiff base transforms into an Amadori product, a steadier and more colorless ketoamine. This stage takes days. Then, the intermediate stage begins, and the Amadori product degrades into colorless or yellow highly unsaturated and prone to polymerization advanced products. The final stage of the reaction continues with dehydration, degradation, oxidation, reduction, condensation and polymerization of compounds, and Amadori products are transformed into highly reactive carbonyl species, such as dicarbonyls and oxoaldehydes. Carbonyl stress can be caused by the accumulation of reactive dicarbonyls, oxoaldehydes or products of glycoxidation or lipoxidation. It is important to note, that the first two stages of Maillard reaction can be reversed, while the final stage is irreversible (4–7).

AGE can be formed within the organism from the non-enzymic glycation of proteins, lipids, and nucleic acids in all body tissues and fluids under physiological conditions, and can be derived from diet and, for example, tobacco smoking as well. It was suggested that the Western diet can enhance the exposure to AGE and their accumulation in the body as a result of overnutrition (8). Despite the fact that AGE are formed as a part of normal metabolism, their overproduction are considered pathogenic. They can alter the structure and function of proteins by cross-linking with them and binding with cell surface receptors, which triggers inflammation and oxidative stress (OS). AGE were shown to be associated with hepatic impairment, neurodegenerative disorder, cancer, diabetic complications, CVD, and the range of other disorders (9).

## 2. AGE receptors

AGEs have several different receptors, but RAGE considered a main. Recognition and binding of AGEs to this receptor, launches the intracellular signaling that disrupts cellular function. RAGE belongs to the immunoglobulin superfamily of receptors. The corresponding gene in human genome is located on chromosome 6 in the major histocompatibility complex between genes for class II and class III. On the promotor of RAGE, there are sites for nuclear factor (NF)-κB, that controls cellular expression of RAGE, and an NF–interleukin-6 (IL-6) DNA binding motif. This associates RAGE with inflammation. Extracellular component of RAGE contains 332–amino acid and consists of two “C”-type domains preceded by one “V”-type immunoglobulin-like domain. RAGE has a single transmembrane domain followed by a highly charged 43–amino acid cytosolic tail. The V domain in the *N*-terminus is important in ligand binding, and the cytosolic tail is critical for RAGE-induced intracellular signaling (10).

Under normal conditions, small amount of RAGE is expressed in vasculature. But in response to accumulation of AGE ligands, RAGE can be upregulated. This upregulation touches on the smooth muscle cells, endothelial cells, and mononuclear phagocytes in diabetic vasculature (11).

Receptors of other types, for example, AGE-R1 (oligosaccharyl transferase-48), -R2 (80K-H phosphoprotein), and -R3 (galectin-3), and the class A macrophage scavenger receptor types I and II, also can recognize and bind AGE ligands. However, they were not demonstrated to transduce cellular signals after engagement by AGEs (12).

## 3. AGEs in atherosclerosis

Under normal physiological conditions AGEs are kept at a low level. However, their overexpression may lead to certain diseases. It is also worth mentioning that while some AGEs support biological functions, other AGEs, usually referred to as toxic AGEs, may be pathogenic and contribute to atherosclerosis and hypertension (13).

Numerous studies in diabetes, which often leads to cardiovascular complications, confirm the association between AGEs and atherosclerosis. Increased AGE levels have been observed in both human diabetes and animal models of the disease, and correlate with the advancement of atherosclerosis (14). Studies have also demonstrated that treatment aimed at AGE reduction or RAGE inhibition can mitigate vascular damage. Non-diabetic atherosclerosis patients have higher AGE-apolipoprotein B (apo B) levels and AGEs have been observed in atherosclerotic plaques in humans as well as in animal models (15, 16).

Increased levels of tissue methylglyoxal and AGEs have been observed in rat models of hypertension, both spontaneous, and sugar-induced. Wistar-Kyoto rats who received methylglyoxal with diet demonstrated elevated tissue AGEs and hypertension. Hypertensive diabetic patients present with higher AGE levels in plasma than non-hypertensive patients (17). A study involving women with preeclampsia–higher blood pressure during pregnancy–revealed higher RAGE expression in vascular tissues. The levels of soluble RAGEs, which suppress AGEs, are inversely associated with the levels of blood pressure in subjects with primary hypertension. AGEs promote stiffening of the vessels by accumulating in long-lived proteins like collagen and elastin and forming protein crosslinks. A recent study in untreated patients with primary hypertension showed an association between elevated AGEs in plasma and vascular stiffness (18, 19). As a result, systolic blood pressure and pulse pressure increase and lead to higher cardiovascular risk. This process is normally observed in aging, however, some conditions associated with elevated AGE expression, such as diabetes, may accelerate it (20, 21). Clinical studies of Alagebrium (formerly known as ALT-711) showed that it broke some protein crosslinks and reduced arterial stiffness in animal models of diabetes, reduced arterial stiffness in aged patients as well as attenuated hypertension and improved endothelial function in subjects with systolic hypertension (22, 23). Several studies involving animal models confirmed that treatment methods aimed at reducing AGE levels also downregulated blood pressure, which again proves the role of AGEs in hypertension (24).

A number of specific AGEs, such as argpyrimidine, glycolaldehyde-pyridine, Nε-carboxy-methyl-lysine (CML), and Nε-carboxy-ethyl-lysine (CEL) have been associated with high blood pressure and atherosclerosis. Additionally, *in vitro* studies of AGEs report that AGE-induced changes in protein functioning correspond to those typical in hypertension and atherosclerosis (25). These complications include endothelial dysfunction, impaired Ca^2+^ metabolism, increased OS, inflammation, and alterations in cell signaling pathways.

## 4. Low-density lipoprotein (LDL) modifications

Over 100 years ago, Nikolai Anitschkow suggested that high total cholesterol levels in blood result in cholesterol deposition in the arterial intima, eventually leading to atherosclerosis. It was later established that lipid accumulation in the vessel cells is not related to total cholesterol levels, but to elevated LDL cholesterol levels as this type of cholesterol has an atherogenic effect (26). A positive association was observed between the atherogenic properties of blood and levels of modified lipids in plasma resulting from an imbalance between modified low-density lipoprotein (LDL) levels and high-density lipoprotein (HDL), the latter having atheroprotective properties (27, 28).

**FIGURE 1:**
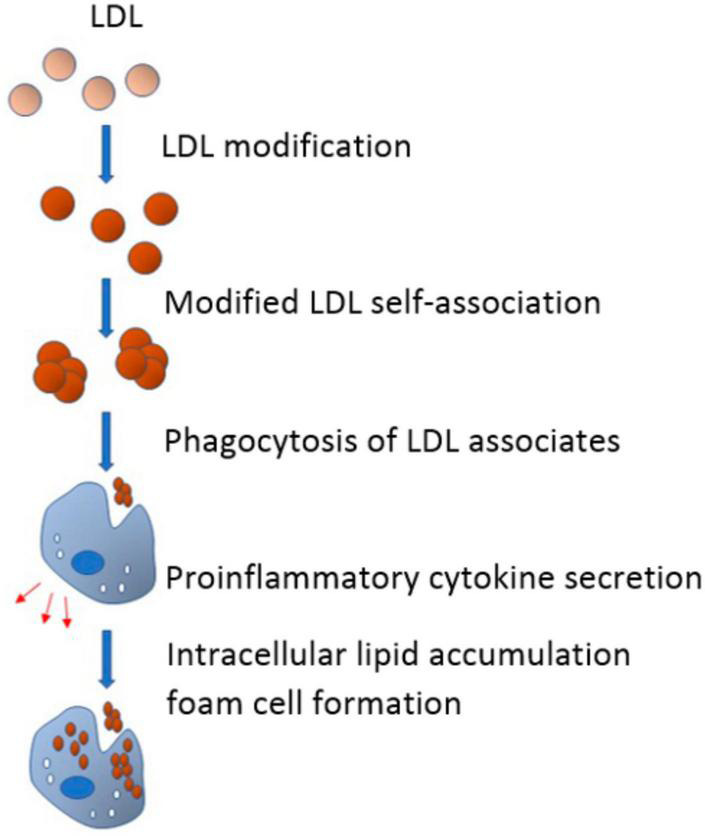
In the blood flow, low-density lipoprotein (LDL) undergoes multiple modifications and acquires atherogenic properties. Modified LDL particles have a tendency to form self-associates that, in turn, promote phagocytosis of subendothelial arterial cells. Being triggered by phagocytosis, proinflammatory response emerges and pro-inflammatory cytokines are secreted. Pro-inflammatory cytokines promote or even cause the accumulation of intracellular lipids, which leads to the formation of foam cells (From 52).

According to the lipid theory of atherosclerosis, serum cholesterol is primarily transported to the arterial cells by LDL. The intracellular lipid accumulation is therefore determined mainly by LDL levels in serum. Non-esterified or “free” cholesterol accounts for almost 50% of the LDL particle weight, making them far more enriched with free cholesterol than other plasma lipoprotein particles. However, not all types of LDL are atherogenic (29). For instance, native LDL cholesterol is not associated with intimal lipidosis, while other LDL subfractions are susceptible to various atherogenic modifications such as desialylation–the earliest and most common modification observed in the blood of subjects with atherosclerosis (30) (see Figure 1).

Sialic acid is an important component of native LDL. It is a carbohydrate found in gangliosides and at the end of biantennary sugar chains in apoB. Following desialylation, galactose–a monosaccharide residue–becomes terminal and exposed externally, as it precedes sialic acid in the carbohydrate chain (31, 32). This allows to isolate desialylated LDL particles from the total LDL using *Ricinus communis* agglutinin I (RCA120) which is very similar to terminal galactose. Once the desialylated and sialylated fractions of LDL were isolated from the total LDL, it became possible to reveal the multiple physicochemical differences between these fractions (33). The study showed that subjects with coronary artery disease (CAD) demonstrated 1.5- to 6-fold higher levels of desialylated LDL, in blood, accounting for up to 60% of total LDL, compared to healthy controls. Noteworthily, some desialylated LDL (around 5–10% of total LDL) can be observed in the blood of healthy people (34). Furthermore, several other chemical and physical modifications of LDL follow desialylation. This sequence of modifications includes decrease in particle size, increase in negative electrical charge and density, lipid loss, and oxidation (i.e., increase in cholesterol covalently bound to apoB) (35).

A number of *ex vivo* studies confirmed the occurrence of these LDL modifications. When native LDL was incubated with serum from patients with atherosclerosis, its desialylation occurred after 1 h. After 3 h, lipid accumulation was observed. Following 6 h of incubation there was a decrease in both the particle size and the ratio of neutral lipids/phospholipids (36). The negative charge increased within 36 h. Following 48–72 h of incubation, the fraction lost tocopherol and became more prone to copper oxidation, eventually leading to an accumulation of lipid peroxidation end products. The study confirmed the main properties of the multiply modified LDL (mmLDL) particles: desialylated LDL particles are smaller and have higher density compared to native LDL. Small dense LDL (sdLDL) has significantly lower sialylation rate, which correlates with its atherogenic properties (27). Additionally, desialylated LDL particles are more electronegative, and this property is positively associated with their desialylation rate, indicating that electronegative [LDL (–)] and desialylated LDL may be parts of the same LDL subfraction. The LDL (–) subfraction in healthy individuals has a high content of desialylated LDL which has lower sialic acid level when compared to native LDL. Furthermore, a study involving LDL (–) and LDL cultured from human aortic intima detected an inverse association between sialic acid levels in the LDL and the particles’ ability to promote intracellular lipid retention (31). The findings confirmed low sialic acid content in such particles, which indicates that they can be considered desialylated lipoproteins. The fact that LDL oxidation normally happens at the later stages of the modification chain indicates that modified LDL is more susceptible to oxidation *in vitro*. However, glycation of apoB lipoprotein in sdLDL particles was observed both *in vitro* and *in vivo*, and the glycated apoB ratio was inversely correlated with the particle size (37).

Interestingly, the artificially generated species of oxidized LDL, observed during numerous *in vitro* experiments, have not been detected in the blood. Although circulating mmLDL particles show some signs of oxidation, it is more probable that the oxidation itself takes place in the vessel intima, rather than in the blood (38). At the same time, a large number of studies carried out during the past 30 years revealed other atherogenic LDL forms circulating in the blood of patients with diabetes and atherosclerosis. These modifications include glycated LDL, desialylated LDL, electronegative LDL, and sdLDL (35).

LDL glycation is a result of chemical modification of apolipoprotein apoB-100 lysine residues by glucose or its metabolites, reacting non-enzymatically with free amino groups of lysine. Studies in patients with diabetes and metabolic syndrome indicate that sdLDL is more prone to glycation which in its turn increases the particles’ susceptibility to oxidation and formation of AGEs, making the LDL more atherogenic (39).

Higher proatherogenicity of sdLDL particles is due to their biochemical and biophysical properties. These particles are smaller and thus can easily invade the arterial cells serving as cholesterol source and promoting lipid accumulation. They have lower affinity for the LDL receptor and as a result–a longer time of circulation in plasma, which makes them more exposed to atherogenic modifications: glycation, desialylation, oxidation, and carbamylation (40). Furthermore, sdLDL induces retention of lipoproteins in the subendothelial area due to its binding affinity to the intimal proteoglycans. Increased sdLDL levels were reported in many atherosclerosis-related conditions, including metabolic syndrome, diabetes, dyslipidemia, and a number of other disorders. Moreover, elevated sdLDL levels were established as an independent predictive factor for related cardiovascular events. The atherogenicity of desialylated LDL can be further enhanced by oxidation (40, 41).

Hence, desialylated LDL particles differ from native LDLs, showing different lipid, carbohydrate, and apoB-100 structure. They are smaller, more electronegative, denser, more susceptible to lipid peroxidation, have lower sialic acid content and are richer in triglycerides and fatty acids.

Glycosylation is another modification occurring to LDL particles. Glycosylation consists in the covalent attachment of glycan moieties to proteins. Glycosylation is presented by following processes: N-glycosylation, which occurs on an asparagine residue (Asn) when present in the motif Asn-X-Ser/Thr (where X is any aminoacid, except proline), O-glycosylation, which occurs on serine (Ser) or threonine (Thr) residues, and addition of long-chain sugars to a core protein forming glycosaminoglycans (42, 43). Proteins that is incorporated into lipoproteins are highly glycosylated. In apoB, apoC-III, apoA-I, and apoE, sialic acid is a predominant terminal residue (44–46). ApoB is a major component of LDL and VLDL structure, which has several potential *N*-linked glycosylation motifs (Asn-X-Ser/Thr), 16 of which are glycosylated. Glycosylation of ApoB is crucial for correct folding, stabilization, and for the assembly and secretion of lipoprotein particles as well.

Glycosylation of ApoE was shown to be an exclusive post-translational modification that occurs in L5 LDL. This contributes to negative surface charge and thus is predicted to interfere with the attraction of LDL to specific targets. L5 appeared to be the atherogenic fraction of human LDL (47).

Since all proatherogenic LDL particles have similar properties, it can be suggested that modified LDLs belong to the same subfraction exposed to multiple modifications (48, 49).

In addition, mmLDL is susceptible to formation and accumulation of large highly atherogenic complexes based on LDL, the so-called self-associates, which play a critical role in intracellular lipid accumulation. Studies have shown that without these self-associates even mmLDL could not induce cholesterol accumulation in vascular cells. Large LDL associates escape the receptor-regulated pathway, resulting in high intracellular cholesterol accumulation (50). A number of trials confirmed that LDL complexes promoted foam cell formation in cultured macrophages by increasing cholesterol ester content. A study in patients with diabetes and CAD established a positive correlation between the atherogenicity of mmLDL and the concentration of LDL self-associates in blood. Another modification which further aggravates lipoprotein accumulation in the vessel walls is the formation of LDL complexes that contain tissue matrix components, including proteoglycans, collagen, elastin, and cellular debris (51, 52).

Furthermore, LDL accumulated in the arterial intima is also exposed to enzymatic changes, as plaque microenvironment promotes the expression of some enzymes, including sphingomyelinase, phospholipase A2, proteases (matrix metalloproteases and cathepsins) as well as cholesterol esterase. Enzymatic modifications promote the development of atherosclerosis by contributing to LDL accumulation and fusion, resulting in its retention in the subendothelial area (53).

The findings of the abovementioned studies indicate that the circulating LDL of patients with atherosclerosis undergoes a sequence of modifications, desialylation being the first of them and the most important for the initiation of atherosclerosis. These changes make the LDL highly atherogenic (54).

It is worth mentioning, that HDL, which is considered to be atheroprotective, can also undergo modifications. These modification affects the properties of particles and include carbamylation, oxidation, and glycation (55).

## 5. Non-enzymatic glycation

High glucose levels in blood can result in glycation of lipids, proteins and nucleic acids *via* non-enzymatic processes. In proteins, the first step is the reaction between glucose and the amino groups of arginine and lysine residues. As a result, an unstable Schiff base is formed which is later transformed into a stable Amadori product (56). Many blood proteins can be affected by glycation, including albumin and immunoglobulins. Structural proteins like collagen who have a long half-life are most susceptible to glycation. The quantification of glycosylated proteins in blood is used as the main method of glycemic control. In lipoproteins, the protein moiety can be also exposed to glycation, affecting their normal function (57).

Non-enzymatic glycation also triggers the generation of oxygen free radicals through a process known as glycoxidation. In this process, molecular bonds are rearranged resulting in a formation of AGEs, which affects the protein functioning irreversibly. As AGE generation requires more time compared to the formation of Amadori products, it usually affects structural proteins. However, there have also been detected AGEs generated from proteins with a shorter lifespan, including apoB in LDL (58).

Another non-enzymatic modification mechanism associated with hyperglycemia is the modification by aldehydes, including MG–a dicarbonyl glucose metabolite with high reducing power. MG quickly reacts with arginine residue of a protein. A heterocyclic compound (hydroimidazolone) is formed as a result of the reaction (59). Thornalley et al. have demonstrated that MG-modified LDL can be observed in elevated concentrations in the blood of diabetic patients. Treatment with metformin decreases MG-LDL levels (60).

## 6. Effects of oxidation and non-enzymatic glycation on lipoprotein function

The research carried out during the past 30 years has established that LDL modification plays a pivotal role in atherogenesis. Although the most knowledge has been gathered around the oxidative modifications, alternative mechanisms involved in the development of the disorder are becoming better and better known. In metabolic syndrome and diabetes, LDL can be affected by various modifications. The oxidatively modified LDL (oxLDL) is a particle that contains pro-inflammatory lipids and protein adducts. Thus, oxLDL is highly atherogenic and contributes to the progression of the disease through various mechanisms (61). Lipid-derived molecules, like malondialdehyde, generated as a result of oxidation, are highly reactive and promote the derivatization of lysine and arginine residues in apoB. As a result, the affinity for the LDL-R is lost and the binding to SR increases. Thus, oxLDL promotes excessive intracellular retention of cholesterol esters by macrophages. Furthermore, the oxidation-induced lipid derivatives generated in oxLDL trigger cytokine, chemokine and growth factor expression in the vessel wall, leading to chronic inflammation and cell proliferation which are typical for atherosclerosis. In addition, the cytotoxic and apoptotic properties of oxLDL stimulate the formation of a necrotic core in advanced plaques. OxLDL fraction includes particles with different oxidation degrees. While minimally oxidized LDL is more pro-inflammatory, extensively oxidized LDL has more capacity to trigger formation of foam cells (62).

It is worth mentioning that glycosylated LDL (gl-LDL), AGE-modified LDL (AGE-LDL), and MG-modified LDL (MG-LDL) are three different groups. Loss of affinity for LDL-R is the main atherogenic feature of gl-LDL. However, it is not as proinflammatory as AGE-LDL which induces both inflammation, apoptosis and foam cell formation since it is generated during oxidative processes. As for MG-LDL, it is smaller in particle size, more prone to aggregation and has higher affinity for binding to intimal proteoglycans (63). Short half-life of LDL circulating in blood has been used as an argument against the possibility of gl-LDL and AGE-LDL formation in blood as protein modification by glucose takes 6–7 days in absence of reducing agents. It was therefore potentially assumed that the formation of modified LDL particles would predominantly take place in LDL retained in injured areas of the arterial wall for a period longer than its plasma lifetime and their presence in the blood would be a reflection of the development of arteriosclerotic lesions. MG-induced modification does not involve glucose. During this modification a highly reactive glucose metabolite reacts with arginine residues of the LDL. Thus, of all the LDL-modifications, this one can be formed within the LDL lifetime in plasma (64).

## 7. Glycation and atherogenesis

Clinical studies have revealed elevated levels of AGE on LDL from patients with diabetes in comparison to healthy controls. AGEs accumulate on long-lived intima proteins with aging. This process is accelerated in diabetes. Non-enzymatic reaction takes place between glucose and proteins/lipoproteins of the arterial wall in diabetes, accelerating the development of atherosclerosis (65). Glucose levels in blood and the time of exposure are the main factors determining the degree of non-enzymatic glycation. Despite the fact that this reaction takes place in all subjects, individuals with diabetes mellitus present with the most adverse effects (66, 67). Also, AGE–apoB levels are up to four times greater in subjects with diabetes. As soon as they have been generated, AGE-protein adducts are practically irreversible (68).

Long term exposure to high glucose levels in blood has been recognized as the main factor leading to complications in patients with diabetes. Hyperglycemia triggers many changes in the artery tissues that can promote atherogenesis. The main pathological mechanisms accounting for the majority of vascular complications in animals and humans with diabetes are the interdependent non-enzymatic glycation of proteins and lipids as well as OS (69).

## 8. Conclusion

Although numerous studies have investigated the nature of atherogenesis and the associated pathogenic processes, some of the mechanisms have not been completely understood yet. The studies carried out during the past three decades have provided ample evidence that LDL modification is the key factor in inducing atherosclerosis. While most research has been carried out around LDL oxidation, this process is closely associated with other modifications. Following an analysis of the available data we concluded that long-term exposure to high glucose levels triggers a chain of LDL modifications enhancing the atherogenicity of the fraction. In this paper we have looked into hyperglycemia-related LDL modifications, including MG-LDL, AGE-LDL, and gl-LDL, and their role in the development of atherosclerosis. Other atherogenic modifications, such as desialylated LDL, electronegative LDL, and sdLDL have also been reviewed. Further studies of multiple modifications are the promising way to expand the understanding of atherosclerosis development and finding potential therapy strategies. Considering glycation, this can be a potential link between atherosclerosis and diabetes melitus, as well as metabolic syndrome.

## Author contributions

AP: writing—original draft preparation. AO: writing—review and editing. All authors contributed to the article and approved the submitted version.
